# Urogenital microbiome, intracellular bacterial communities, and their contribution to urinary tract infections

**DOI:** 10.1128/spectrum.01247-25

**Published:** 2025-10-07

**Authors:** Luciana Robino, Nicolás Navarro, Nicole Canales-Huerta, María José González, Erlen Cruz, Rafael Sauto, Cecilia Morales, Florencia Neffa, Javier Zeballos, Gerdhard L. Jessen, Pablo Zunino, Paola Scavone

**Affiliations:** 1 Unidad Académica de Bacteriología y Virología, Facultad de Medicina, UdelaR, Instituto de Higiene 113078 , Montevideo, Uruguay; 2 Laboratorio de Biofilms Microbianos, Depto. de Microbiología, Instituto de Investigaciones Biológicas Clemente Estable 113067, Montevideo, Uruguay; 3 Servicio de Urología, Hospital Maciel, Montevideo, Administración de Servicios de Salud del Estado 419193, Montevideo, Uruguay; 4 Instituto de Ciencias Marinas y Limnológicas, Universidad Austral de Chile 28040, Valdivia, Chile; 5 Depto. de Microbiología, Instituto de Investigaciones Biológicas Clemente Estable, Montevideo, Uruguay; Quest Diagnostics Nichols Institute, Chantilly, Virginia, USA

**Keywords:** urinary microbiome, EQUC, intracellular bacterial communites, urinary tract infection

## Abstract

The urinary tract was long considered a sterile environment, but recent studies have revealed the presence of diverse microbial communities in healthy individuals collectively known as the urinary microbiome or urobiome. Although the urobiome has been studied primarily in populations from North America, Europe, and Asia, little is known about its characteristics in Latin America. In this work, we conducted a descriptive analysis of the urobiome of 77 adults, 40 females, and 37 males with and without symptoms of urinary tract infections (UTI) from Uruguay using a strategy that includes expanded quantitative uroculture, 16S rRNA gene sequencing from urine, and intracellular bacterial community evaluation. The microbial profiles revealed a wide diversity in the bacterial community composition, including both *Lactobacillus*-dominated and diverse non-*Lactobacillus* communities. Notably, several genera frequently associated with health or disease, such as *Gardnerella*, *Staphylococcus*, *Streptococcus*, and *Escherichia*, were detected in many samples. We also observed associations between community types and host factors, such as gender, presence of symptoms, and IBC. These findings contribute to expanding our understanding of the human urobiome and highlight the need to consider geographic and cultural diversities when characterizing microbial communities and their implications for health.

**IMPORTANCE:**

The concept of a sterile urinary tract has been challenged by the discovery of complex microbial communities in urine samples from healthy individuals. Most existing studies on the urinary microbiome focus on populations in the Global North, resulting in limited data from Latin America. Here, we present the first study characterizing the urobiome of an adult Uruguayan population using 16S rRNA gene sequencing. By analyzing associations between bacterial community types and individual metadata, we provide insights into the diversity and structure of urinary microbiota in a previously understudied population. These results broaden the current understanding of the urobiome and emphasize the importance of including diverse populations in microbiome research to achieve a more comprehensive and representative view of microbial contributions to health and disease.

## INTRODUCTION

Humans are a composite organism (holobiont) that lives in a dynamic state with a diversity of microorganisms. The set of microbes and their genetic material that inhabit different organs and systems is called the microbiome. Since the beginning of the human microbiome project in 2009, there have been enormous advances in our knowledge of the human microbiome (1). The vast majority of studies have focused on the intestinal microbiota, understanding its role in various physiological and immunological functions (2). The urinary tract was not included in the original human genome project because it was considered a sterile anatomical site. Applying culture-independent studies to urine samples from asymptomatic individuals has demonstrated that the urinary tract is not sterile and is composed of a wide array of microorganisms, including bacteria, viruses, and fungi (3, 4), called the urobiome (5). Although there have been several publications on the composition of the urobiome in different health and disease states, it is still not known whether there is a particular cluster of microorganisms associated with one situation or the other.

Another relevant aspect is that some bacteria that are part of the urobiome can be found intracellularly within the bladder epithelial cells, and this needs to be taken into account when studying the urinary microbiome (6).

Culture-independent methods, such as 16S ribosomal RNA (rRNA) amplicon sequencing, can reveal the composition and community structure of the urobiome. However, this method cannot distinguish between living and dead microorganisms or identify their physiology and function. Thus, expanded quantitative urine culture (EQUC) is a quantitative culture method used to complement 16S rRNA gene sequencing (7).

This study aimed to describe the microorganisms present in the urogenital tract of healthy adults and persons with suspected UTI using three different approaches. All samples were subjected to 16S rRNA gene sequencing, EQUC, and IBC identification and characterization through confocal microscopy. This multiple-parameter study brings new insight into the role of urobiome in a population-less study as Latin American. It is necessary to expand the knowledge in order to determine their contribution to health and disease. So far, this is the first study that includes IBC in the urobiome.

## MATERIALS AND METHODS

### Population

Two Uruguayan adult groups (>18 years old) were voluntarily enrolled in the present study from 2019 to 2022. The “asymptomatic” or control group comprised people without any urinary tract infection symptoms/urological pathologies/antibiotic treatments in the last month before obtaining the sample. The symptomatic group included patients with signs and symptoms of UTI who were treated at the Polyclinic of Urology of the Hospital Maciel or Centro Hospitalario Pereira Rossell in Uruguay. All the participants signed an informed consent that was included in the evaluation, and a number was assigned in order to anonymize the samples. All the procedures were evaluated and approved by the Ethical Committee in Human Research from IIBCE (CEI-IIBCE-004) and UdelaR (Exp No. 070153-000360-19). The research follows the national regulations in human research.

### Urogenital samples

Clean midstream urine samples were self-collected in 120 mL sterile polypropylene containers and stored immediately in the fridge (4°C) in the laboratory; a number was assigned to preserve the identity of the participants. All procedures were done in the following 24 h. Midstream urine collection was chosen and approved by the ethics committee as the preferred method specifically to avoid subjecting healthy volunteers to invasive procedures. Given that this method of urine collection may include microorganisms from the urethral or genital tract, the use of the term 'urogenital sample' is recommended (8).

### Expanded quantitative urine culture (EQUC)

One hundred microliters of urine was plated in different media and environmental conditions: (A) McConkey lactose agar and blood agar 24–48 h, 37°C; (B) blood agar and chocolate agar, 24–48 h, 37°C, 5% CO_2_; and (C) anaerobic blood agar and Man Rogosa Sharpe (MRS) agar, 24–72 h, 37°C, 10% CO_2_ (AnaeroGEN 2.5 L, Thermo Scientific). However, in the present work, we used EQUC to detect low-abundance species rather than to measure CFUs. Once colonies were observed on the surface of the plates, they were spotted again in the same condition for preservation at −80°C and identification through matrix-assisted laser desorption/ionization (MALDI-TOF) (Bruker Daltonik Microflex).

### Intracellular bacterial communities (IBC) in urine

IBC were determined by direct visualization of cytocentrifuged urine. Accordingly, 1 mL urine was subjected to cytocentrifugation (380 × *g*, 6 min) to obtain the desquamated cells and fixed with 4% PFA. The slides were stained with anti-uroplakin III (UPIII, Santa Cruz Biotechnology), wheat germ agglutinin (WGA, molecular probes), and Hoechst 33341 (molecular probes) as described before and observed with a confocal scanning light microscope (CSLM). Also, in order to recover and identify intracellular bacteria, a gentamicin treatment was performed to kill all the extracellular bacteria (6). Briefly, 14 mL of urine was cytocentrifuged at 95 × *g* for 5 min at RT; the supernatant was discharged; and the cells were subjected to treatment with gentamicin for 2 h. Afterward, cells were lysed with Triton X-100 to recover the intracellular bacteria and identify them using MALDI-TOF as described before (6).

### DNA extraction

In order to obtain DNA from all the microorganisms present in the sample, 5 mL of urine was centrifuged at 8,000 rpm for 15 min. The supernatant was discharged, and the pellet was resuspended in 250 µL of PBS. The samples were subjected to DNA extraction using the automatic Pure Prep32 (Molgen) and following the instructions of the PurePrep Pathogen DNA Extraction Kit (Molgen). DNA quantification was performed using the Qubit dsDNA HS Assay Kit (Thermo Scientific).

### 16S rRNA sequencing and processing

16S rRNA gene sequence libraries were prepared with the 16S Rapid Sequencing Amplicon Barcoding Kit (Oxford Nanopore Technologies—ONT, Oxford, UK, SQK-16S024) according to the standard procedures described by the ONT. The complete 16S rRNA gene was amplified using 10 µL input DNA purified from urine samples, LongAmp Taq 2× Master Mix (New England Biolabs, Ipswich, MA, USA) and the barcoded nanopore sequence primers included in the kit. The DNA amplification was performed on a Swift ProGene real-time PCR thermal cycler (ESCO) using the program; 1 min denaturation at 95°C, 35 cycles (95°C—20 s, 55°C—30 s, 65°C—2 min) and a final extension step of 5 min at 65°C. The 16S rRNA gene amplicons were quantified using Qubit dsDNA HS Assay Kit (Thermo Scientific). Equal amounts of amplicons per sample were pooled, and the library was further processed as described by the manufacturer with some modifications. First, electrophoresis gels were performed to visualize the PCR results. Next, purification of the mixed library was performed using the automated Pure Prep32 system with the PurePrep Pathogen DNA Extraction Kit (Molgen). After that, quantification was done again to check the amount of DNA. All barcode libraries were pooled at a ratio of 50–100 fmoles in 10 µL of 10 mM Tris-HCl pH 8.0 with 50 mM NaCl. Next, 1 µL of RAP was added to the barcodes and incubated for 5 min at RT. The library was used for loading into the MinION Flongle Flow Cell (version FLO-FLG0001). The flow cells were primed with a mix of Flush Buffer and Flush Tether. After that, 5 µL of DNA library was mixed with 15 µL of sequencing buffer II and 10 µL of loading beads II. Sequencing was performed using MinIon (Oxford Nanopore Technologies—ONT, Oxford, UK) for approximately 24 h. All sequence data were uploaded in the National Center for Biotechnology Information's Sequence Read Archive under the BioProject ID PRJNA1247451.

The taxonomic identification of bacteria present in the urine of donors was performed using the Metagenomics workflow ("What’s in my pot?") (https://github.com/epi2me-labs/wf-metagenomics) from EPI2ME (Oxford Nanopore Technologies—ONT, Oxford, UK), which enables direct taxonomic classification from unassembled reads generated by MinKNOW. To enhance the accuracy of taxonomic assignment, sequences were filtered using Minimap2 v2.26-r1175. The obtained results were downloaded in a comma-separated value (CSV) file, identifying the bacterial genera and species present in each sample. Then, the results in the CSV file of the EPI2ME 16S workflow output were used for further analysis using R (Rstudio v 4.3.3, R Core Team 2024).

### Statistics

Alpha diversity, observed richness, Shannon, and inverse Simpson diversity indices were calculated using vegan package on R (R Core Team 2024) (9). A Welch’s *t*-test was used to test for significant differences in richness while Kruskal-Wallis was used for Shannon and inverse Simpson diversity indices. Differential abundance analysis was tested using the DESeq2 package (10). Co-occurrence networks were constructed using the NetCoMi package (11). Further filtering was carried out using the Phyloseq package in R, and graphs were created with the ggplot2 package (12).

## RESULTS

### Population

A total of 77 adults participated in the present study, that is, 40 females (mean age 43.3 +/− 10.0) and 37 males (mean age 48.0 +/− 14.8) (Table 1). Each population was also divided according to the presence of symptoms of UTI (22 females mean age 47.2 ± 6.65/17 males mean age 56.5 ± 14.6) and without symptoms or the control group (18 females mean age 39.9 +/− 13.1 and 20 males mean age 40.8 +/− 10.9). Elderly people were considered >60 years, and, in this group, we have seven males (six with symptoms, mean age 69.8 +/− 6.65) and five females (mean age 62.8 ± 2.77). As we did not have records about menopausal status, we decided to stratify this population when >55, and we observed six cases, three with UTI symptoms and three without, but all of them with IBC.

**TABLE 1:** Demographic description of the population involved in the study

Demographics	
Age	
Mean (SD)	45.8 ± 12.9
Median (min, max)	45 (23, 83)
Sex	
Female (*N*, age +/− SD)	40; 43.3 ± 10.0
Male (*N*, age +/− SD)	37; 48.0 ± 14.8
Total	77
Females (*N*, age +/− SD)	
Symptoms	22; 47.2 ± 6.65
IBC	24; 47.2.0 ± 10.6
Symptoms and IBC	15; 48.0 ± 7.67
Postmenopausal (>55)	6; 61.5 ± 4.03
Elder (>60)*	5; 62.8 ± 2.77
Males (*N*, age +/− SD)	
Symptoms	17; 56.5 ± 14.6
IBC	17; 51.8 ± 20.2
Symptoms and IBC	10; 62.1 ± 13.9
Elder (>60)	7; 69.8 ± 6.65

### EQUC

**Fig 1:**
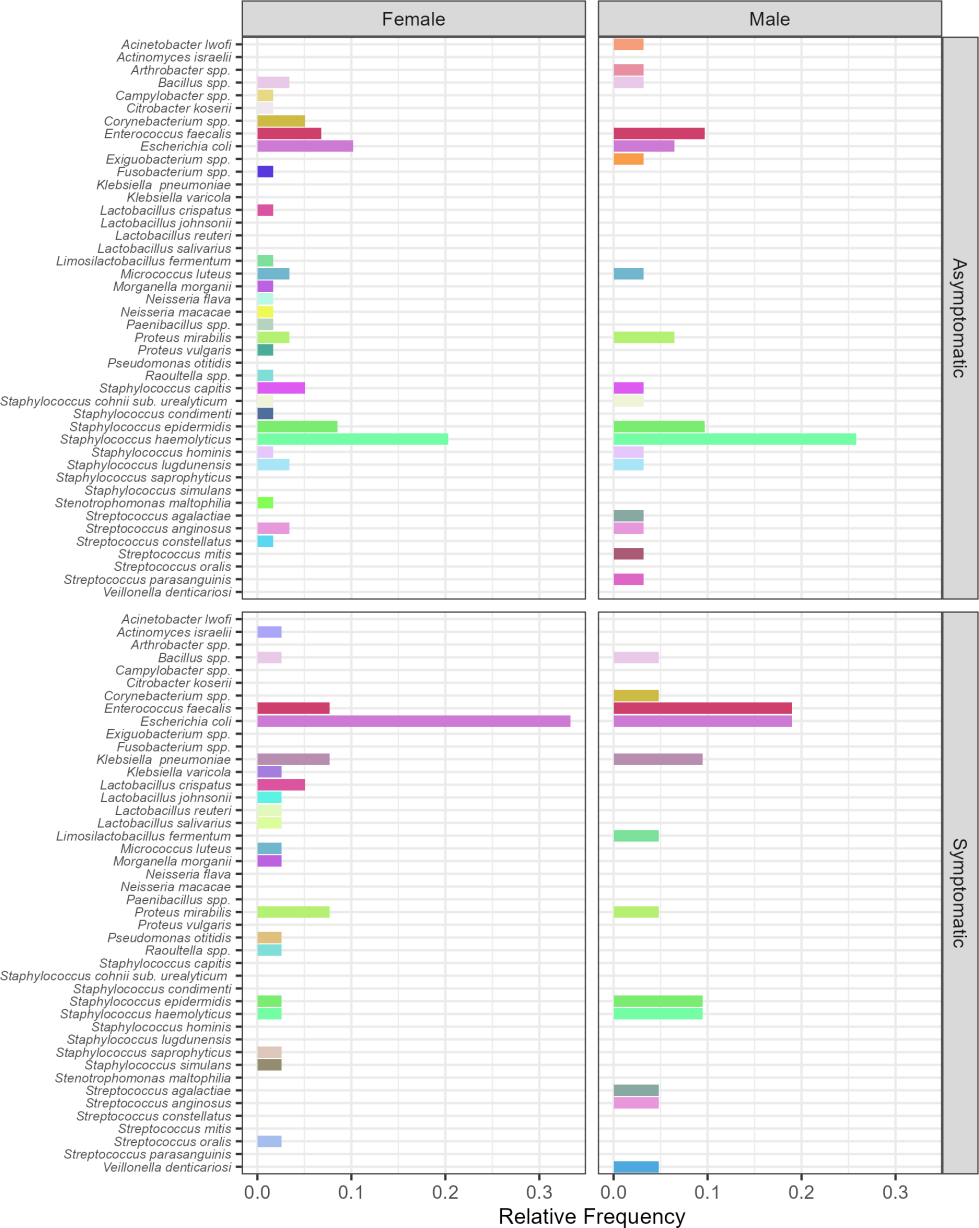
Relative frequency of the microorganism identified by MALDI-TOF and recovered through EQUC in the entire population (female and male) according to the presence or absence of symptoms.

The expanded urine culture revealed some differences among the microorganisms isolated from males and females. The most frequent microorganisms in males were *Staphylococcus haemolyticus*, *Enterococcus faecalis*, *Staphylococcus epidermidis*, *Escherichia coli*, and *Proteus mirabilis* (relative frequencies of 0.22, 0.18, 0.09, 0.09, and 0.065, respectively, Fig. 1). In asymptomatic males, *S. epidermidis* and *S. haemolyticus* were predominant, while in symptomatic males, *E. faecalis* and *E. coli* were more frequently observed (Fig. 1).

In the female population, the most frequent microorganism was *E. coli*, followed by *S. haemolyticus*, *E. faecalis*, *S. epidermidis*, and *P. mirabilis* (relative frequencies of 0.19, 0.13, 0.07, and 0.06, respectively). In asymptomatic females, a great diversity of *Staphylococcus* spp. was observed with predominance of *S. haemolyticus* and *S. epidermidis*, as observed also in asymptomatic males. In symptomatic females, *E. coli* was the most frequent microorganism isolated, followed by *E. faecalis* and *P. mirabilis*.

The EQUC allows the recovery of a great diversity of microorganisms that are not recovered with the normal urine culture, as observed in the female population with *Lactobacillus* spp. (relative frequency of all species 0.071) and different *Staphylococcus* species (relative frequency of 0.305). These two populations were scarcely recovered from male urine. However, in the male population, the proportion of *S. haemolyticus* was higher than in females, but the range of diversity of other species was lower. On the contrary, more *Streptococcus* spp. were recovered in male urine as other species that were present only in males, such as *Acinetobacter iwoffii*, *Arthrobacter* spp., *Bacillus pumilus*, *Exiguobacterium* spp., *Micrococcus luteus*, and *Veillonella denticariosi* (Fig. 1).

### IBC

**Fig 2:**
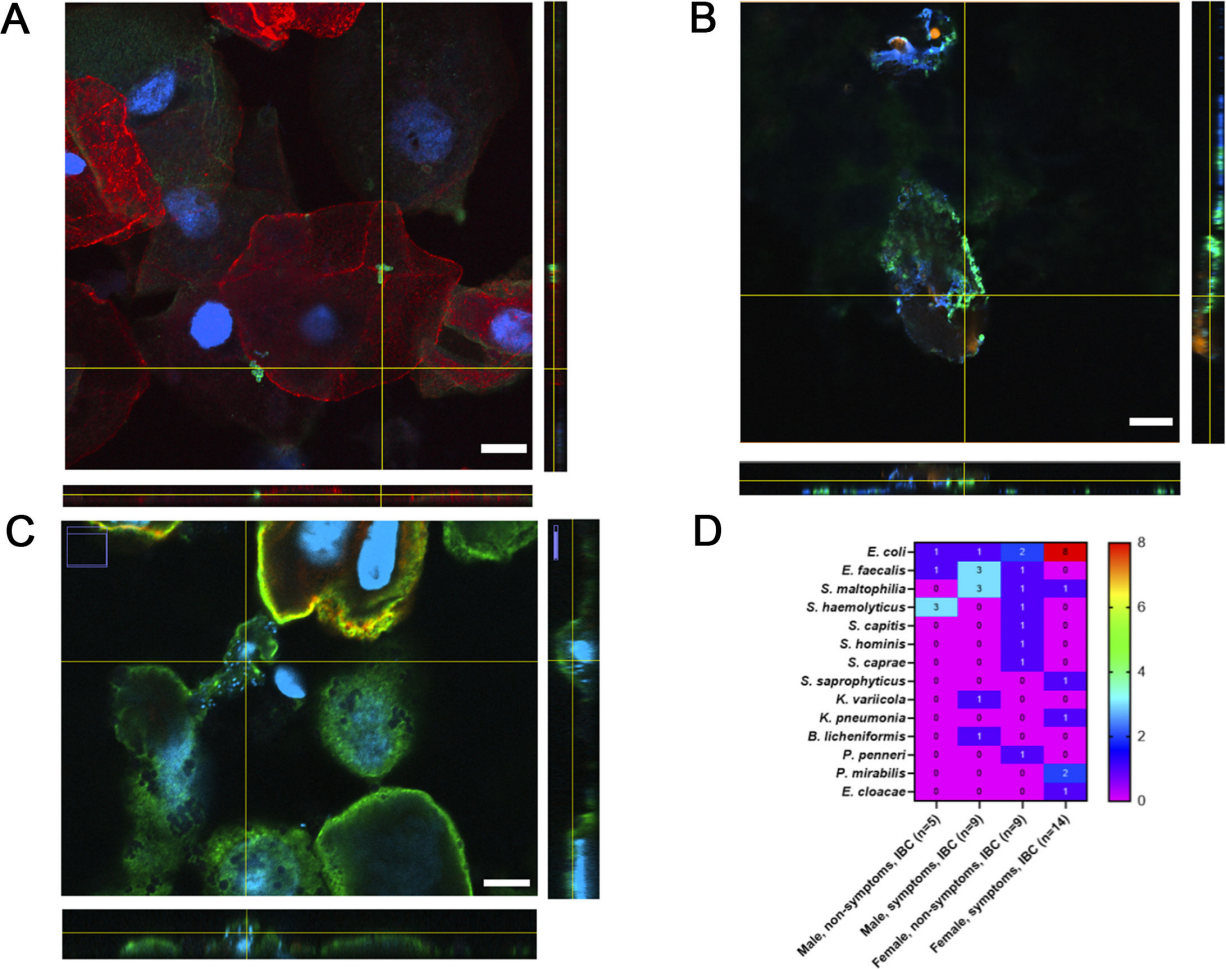
(**A–C**) CSLM representative images of *E. faecalis* (A) and two bacilli *E. col*i (B) and *S. maltophilia* (C) in the desquamated cell from urine. The colors represent in red uroplakin III, green wheat germ agglutinin, and blue Hoescht 33341. The scale bar represents 10 µm. (**D**). Frequency distribution of different intracellular etiological agents detected in individuals with and without symptoms. The heatmap displays bacterial species on the *y*-axis and participant groups on the *x*-axis, which are categorized by sex and symptom presence. The color gradient represents the number of occurrences ranging from 0 (purple) to 8 (red).

A culture-dependent methodology was used to isolate and identify intracellular bacteria that were also visualized and confirmed by CSLM (Fig. 2A through C). Intracellular bacteria were detected in 14 females with symptoms, that is, nine females with non-symptoms, nine males with symptoms, and five males with non-symptoms, representing 48% of the total adults. The remaining 52% of the population without intracellular bacteria was divided into eight females with symptoms, nine females with non-symptoms, eight males with symptoms, and 15 males with non-symptoms (Table S1).

The most frequently intracellularly detected species include *E. coli*, *S. maltophilia*, *S. haemolyticus*, and *E. faecalis*, with notable variations between groups (Fig. 2D). For example, *E. coli* was found more frequently in symptomatic individuals, particularly in females. *S. maltophilia* and *S. haemolyticus* were more prevalent in males with and without symptoms, respectively. Several other species, such as *S. saprophyticus* and *P. mirabilis*, appeared less frequently and in specific groups. These findings suggest potential differences in the intracellular bacterial community between symptomatic and asymptomatic individuals and females vs males.

The IBC etiological agents in symptomatic females were mainly *E. coli* (8/14 cases, 57%), followed by *P. mirabilis* (2/14, 14%), *S. maltophilia*, *S. saprophyticus*, *K. pneumoniae*, and *E. cloacae* (1/14, 7% each, Fig. 2D). In the case of asymptomatic females with IBC, the etiological agents were more diverse. *E. coli* was only detected in two cases, and the others were observed only in one case each (*E. faecalis*, *S. maltophilia*, *S. haemolyticus*, *S. capitis*, *S. hominis*, *S. caprae*, and *P. penneri*). In symptomatic males with IBC, *E. faecalis* and *S. maltophilia* were the most frequent etiological agents (three cases each, 3/9, 33%). Then *E. coli*, *K. variicola*, and *B. licheniformis* were recovered in one case each. Finally, in the group of asymptomatic males with IBC, *S. haemolyticus* was recovered in three cases (3/5, 60%), followed by *E. coli* and *E. faecalis* (one case each).

### Urogenital microbiome diversity

To evaluate urogenital microbiome diversity across the study population, we assessed alpha diversity using observed richness, the Shannon index, and the inverse Simpson index.

**Fig 3:**
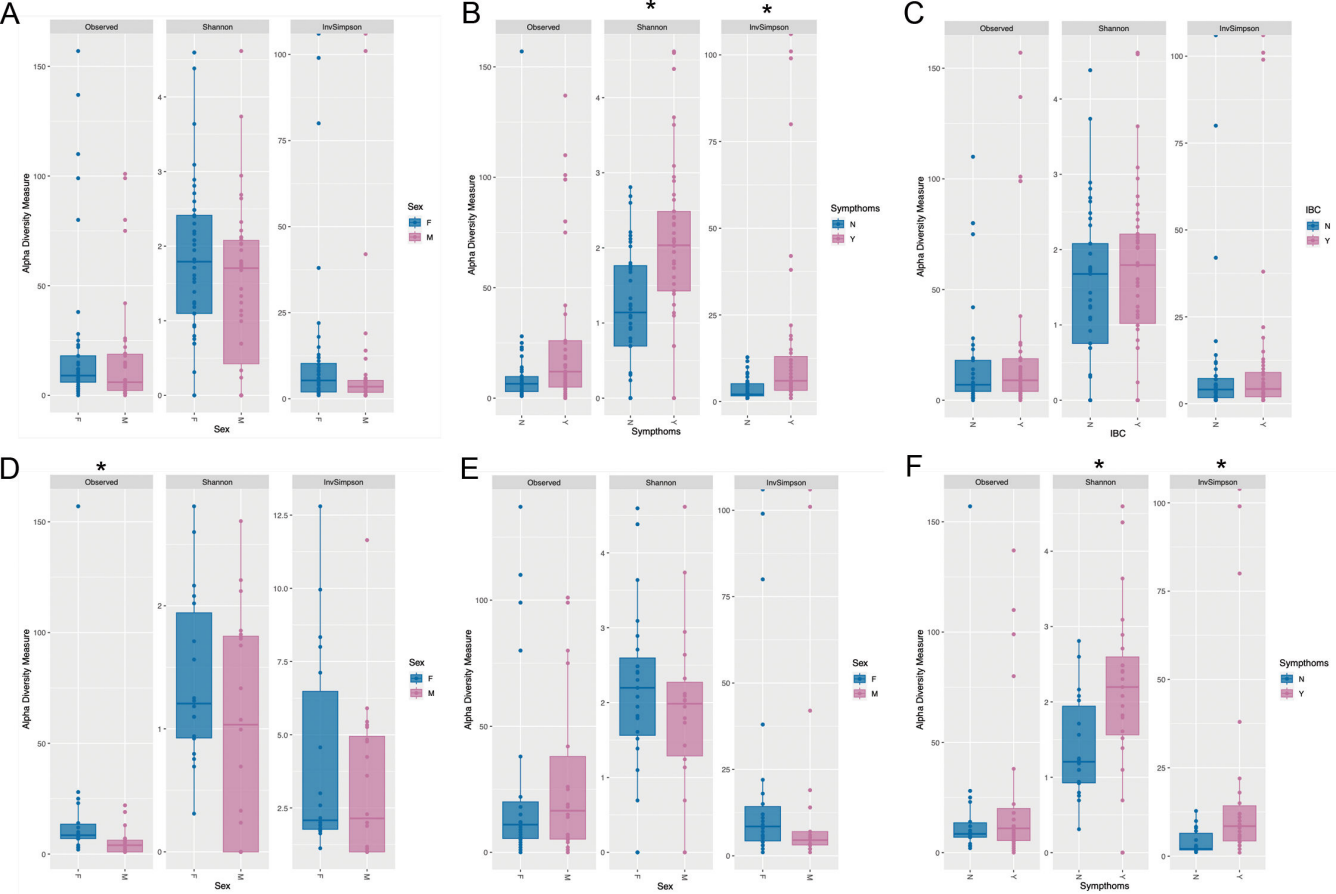
Alpha diversity comparison: (**A**) in all the populations according to gender, F = female and M = male; (**B**) in all the populations according to the presence of symptoms (Y) and non-symptoms (N); (**C**) in all the populations according to the presence of intracellular bacterial communities (Y) or absence (N); (**D**) in the population without symptoms and according to gender, F = female and M = male; (**E**) in the population with symptoms and according to gender, F = female and M = male; and (**F**) female population with and without symptoms. * depicts the cases where significant differences were obtained (*P* < 0.05).

No significant differences were found between all males and females (Fig. 3A). However, when comparing symptomatic and asymptomatic individuals, significant differences were observed across multiple diversity indices, with symptomatic individuals exhibiting significantly higher richness and diversity (observed richness: *P* < 0.01; Shannon index: *P* < 0.001; inverse Simpson index: *P* < 0.0001) (Fig. 3B).

In contrast, no significant differences were detected when comparing individuals with and without IBC (Fig. 3C). Among asymptomatic individuals, females exhibited a significantly higher richness compared to males (*P* < 0.01) (Fig. 3D), while no significant differences were observed between symptomatic males and females (Fig. 3E).

Finally, when comparing females with and without symptoms, significant differences were found in the Shannon and inverse Simpson indices (*P* < 0.01 and *P* < 0.0001, respectively) (Fig. 3F). These results suggest that urogenital microbiome diversity is influenced by symptom status but not by sex alone or the presence of intracellular bacterial communities.

### Urogenital microbiome composition

**Fig 4:**
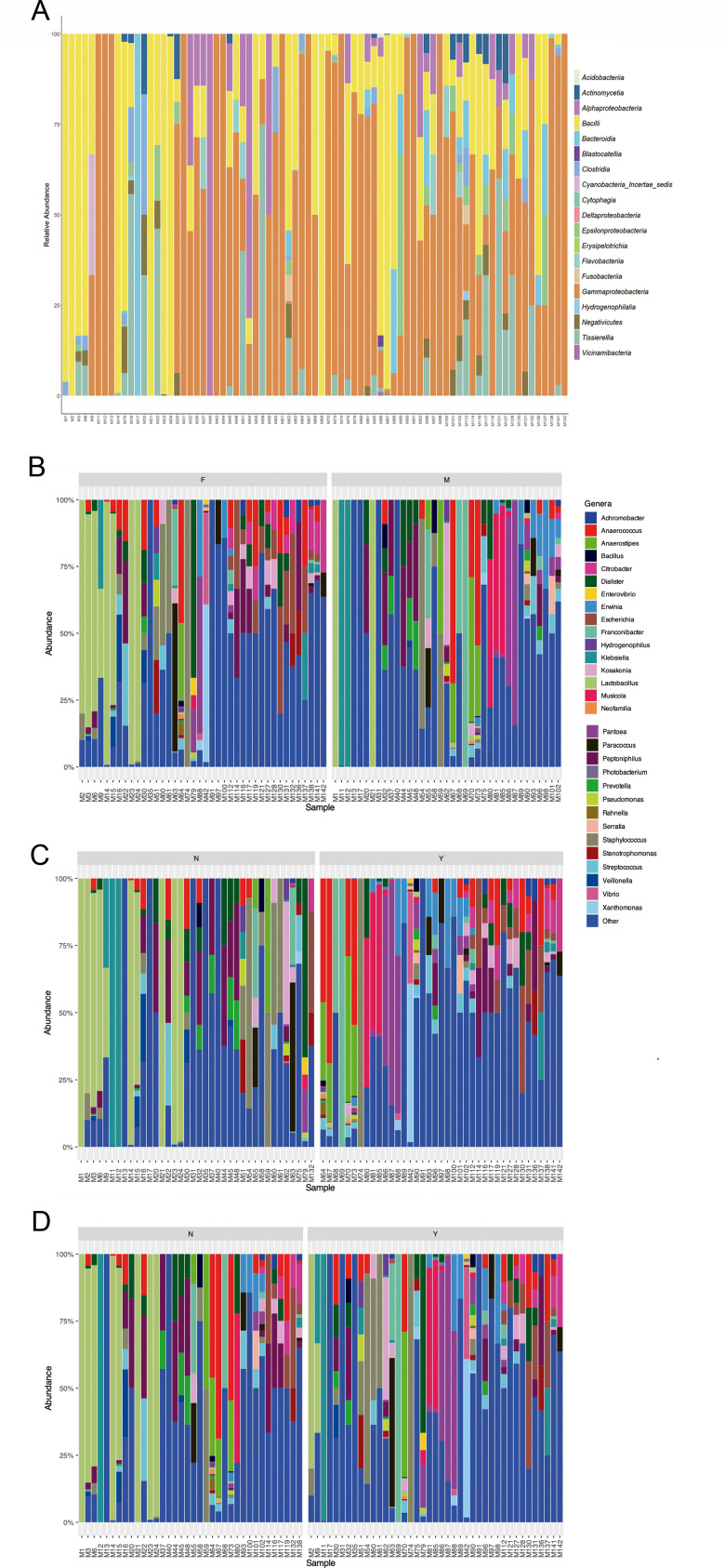
(**A**) Urogenital microbiome composition of the entire population. (**B**) Urogenital microbiome composition of females and males. (**C**) Urogenital microbiome composition in the population according to the presence (Y) or absence (N) of symptoms. (**D**) Urogenital microbiome composition in the population according to the presence (Y) or absence (N) of IBC.

**Fig 5:**
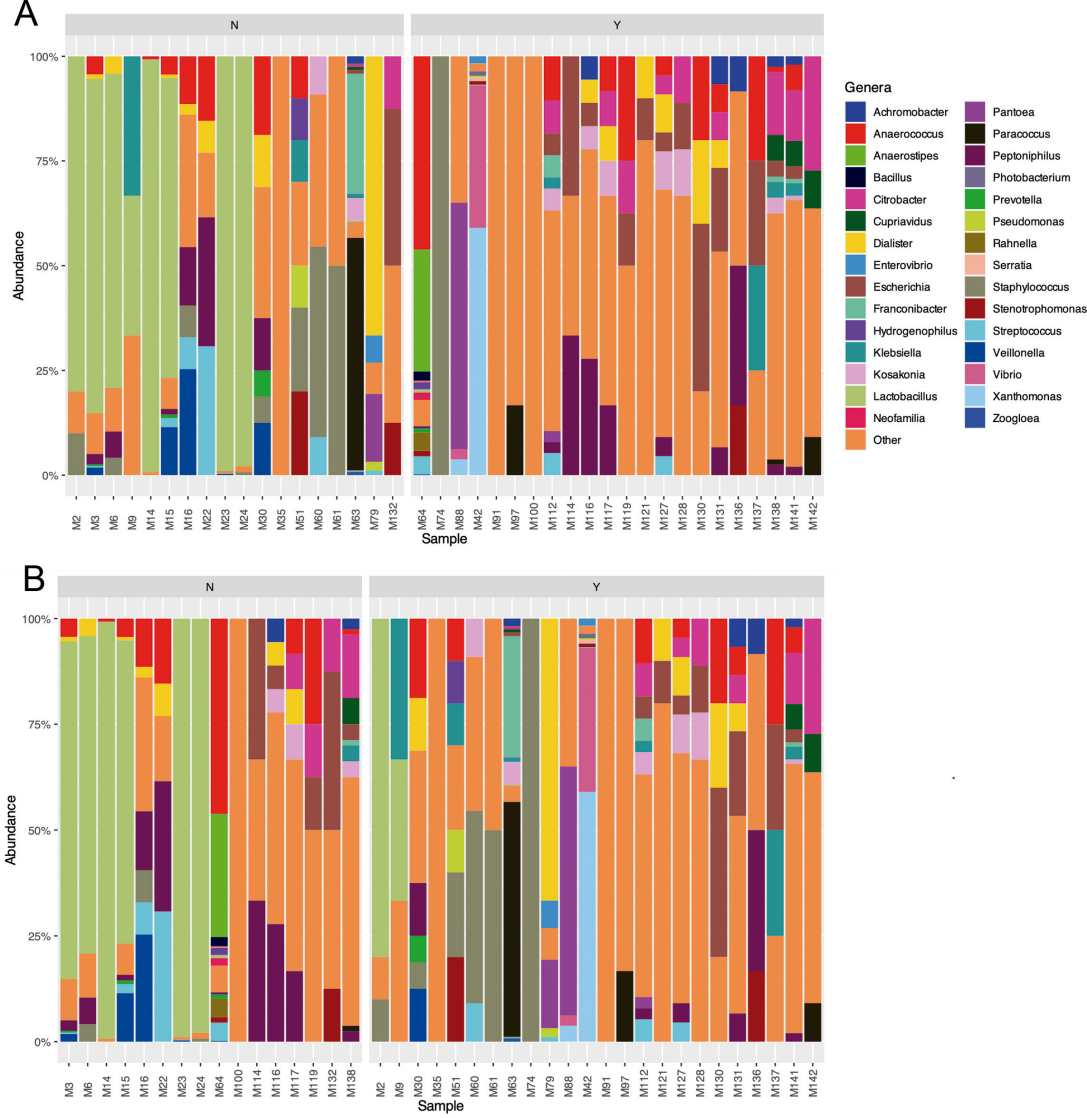
(**A**) Female urogenital microbiome composition in the population with (Y) and without (N) symptoms. (**B**) Female urogenital microbiome composition in the population with (Y) and without (N) IBC. The top 30 genera are presented in different colors.

The composition of the urogenital microbiome in all samples is shown in Fig. 4. In the entire female population, *Lactobacillus crispatus*, *Lactobacillus iners*, *Dialister propionicifaciens*, *Escherichia coli*, *Enterobacter* spp., and *Staphylococcus* spp. were the most abundant microorganisms (Fig. 4B and 5A). In females without symptoms, *L. crispatus* and *L. iners* were present in a higher proportion, while components of this genus disappeared in the case of females with UTI symptoms (Fig. 5A). *E. coli* was the most abundant genus in females with symptoms, followed by *Enterobacter* spp. (Fig. 5A). In the male population, other species were observed compared with females, such as *Musicola* spp., *Peptoniphilus* spp., *Pantoea* spp., and *Dialister* spp. (Fig. 4B). In males without symptoms of UTI, *Peptoniphilus coxii* and *L. iners* were abundant, while in males with symptoms *Musicola paradisiaca*, *Pantoea delayi*, *Anaerostipes* spp., and *Anaerococcus* spp. were observed (Fig. 5B).

**Fig 6:**
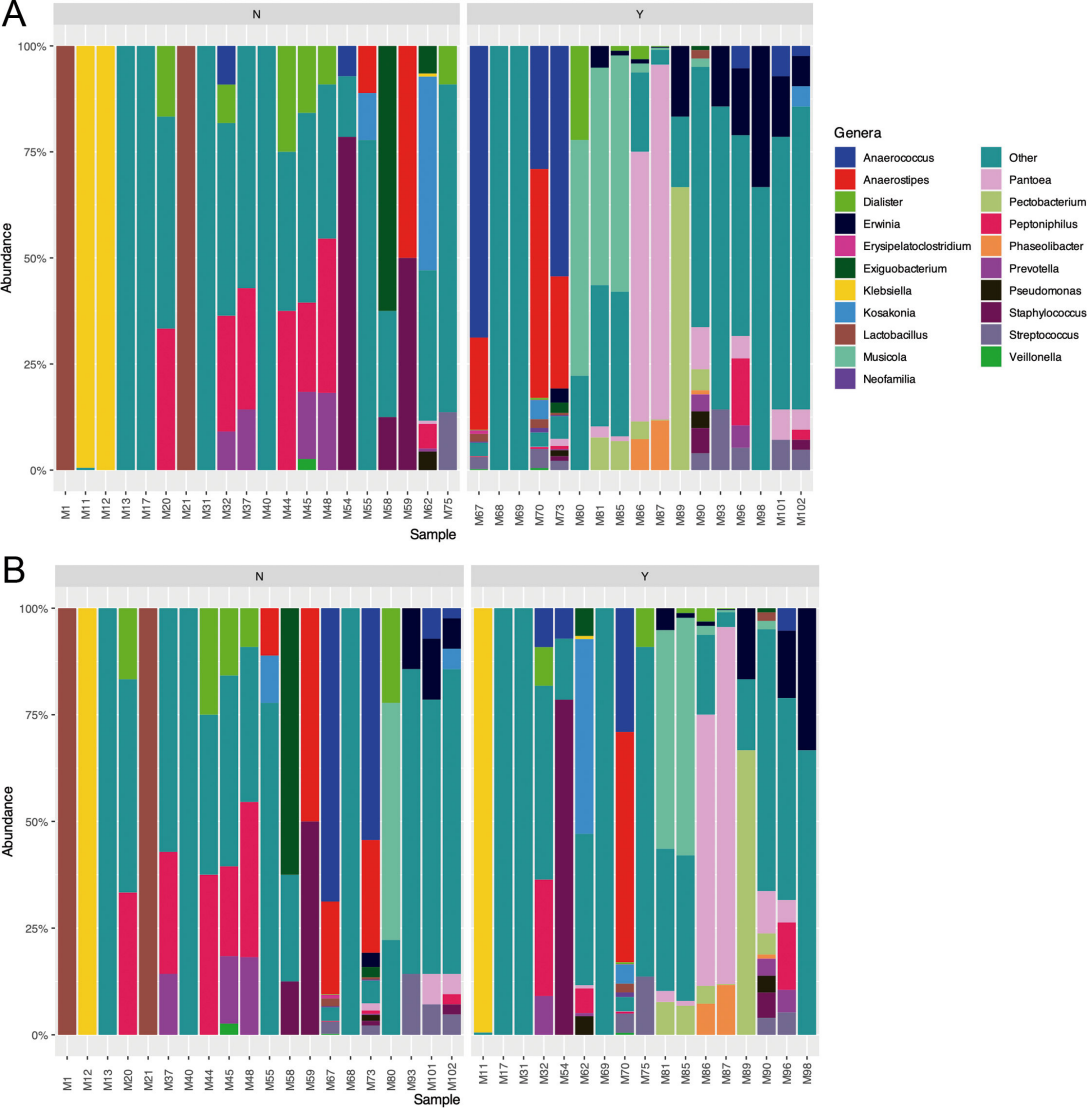
(**A**) Male urogenital microbiome composition in the populations with (Y) and without (N) symptoms. (**B**) Male urogenital microbiome composition in the population with (Y) and without (N) IBC. The top 30 genera are presented in different colors.

When the urogenital microbiome was analyzed regarding the presence or absence of IBC (Fig. 6A and B), *L. crispatus*, *Enterobacter cloacae*, *L. iners*, *Enterobacter sichuanensis*, and *E. coli* were the most abundant genera in females without IBC. In the case of females with IBC, *Dialister* spp., *L. iners*, *E. coli*, and *Staphylococcus* spp. were the main genera. In males without IBC, *L. iners* was also observed, but *Peptoniphilus* spp., *Anaerococcus* spp., and *Anaerostipes* spp. were the more abundant taxa in this group of individuals. In the male population with IBC, *Musicola* spp., *Pantoea* spp., *Yokonella* spp., *Erwinia* spp., and *Peptoniphilus* spp. were observed, among others.

The analysis of urogenital microbiome composition across different ages revealed differences associated with both age and the presence of symptoms (Table S2). Among females younger than 55 years without symptoms, the microbiome was dominated by the class *Bacilli* (relative abundance 39.4%) with *Lactobacilli* (*L. crispatus* and *L. iners*) being the most abundant species. In contrast, symptomatic females showed a decrease in the class *Bacilli* and an increase in *Gammaproteobacteria* that include members of *Enterobacterales* (*Escherichia* 13.5%) and *Burkholderiales* (*Achromobacter* and *Ralstonia* 15.7%). In postmenopausal (consider >55 years old), the sample size was limited to six cases (three symptomatics and three non-symptomatic). In this group, the microbiome was also dominated by *Gammaproteobacteria*, with *Ralstonia* spp., *Strenotrophomonas* spp., *Escherichia* spp., *Achromobacter* spp., and *Enterobacter* spp. as the most abundant. In the class *Bacilli*, *Lactobacillus crispatus* is the most abundant and represented species.

In asymptomatic males, the microbiome showed other classes besides *Bacilli* and *Gammaproteobacteria*, such as *Alphaproteobacteria* and *Bacteroidia* (Table S3). In symptomatic males, *Gammaproteobacteria* (44.8% relative abundance) and *Bacilli* increase with the appearance of *Bacillus* in addition to *Lactobacillus* when compared with asymptomatic males. In the elderly male group (>60 years, *n* = 7, six symptomatic, all of them with IBC), the most abundant class was again *Gammaproteobacteria*, with orders, such as *Burkholderiales*, *Enterobacterales*, and *Xanthomonadales*, featuring prominently among the top 10 most abundant taxa.

**Fig 7:**
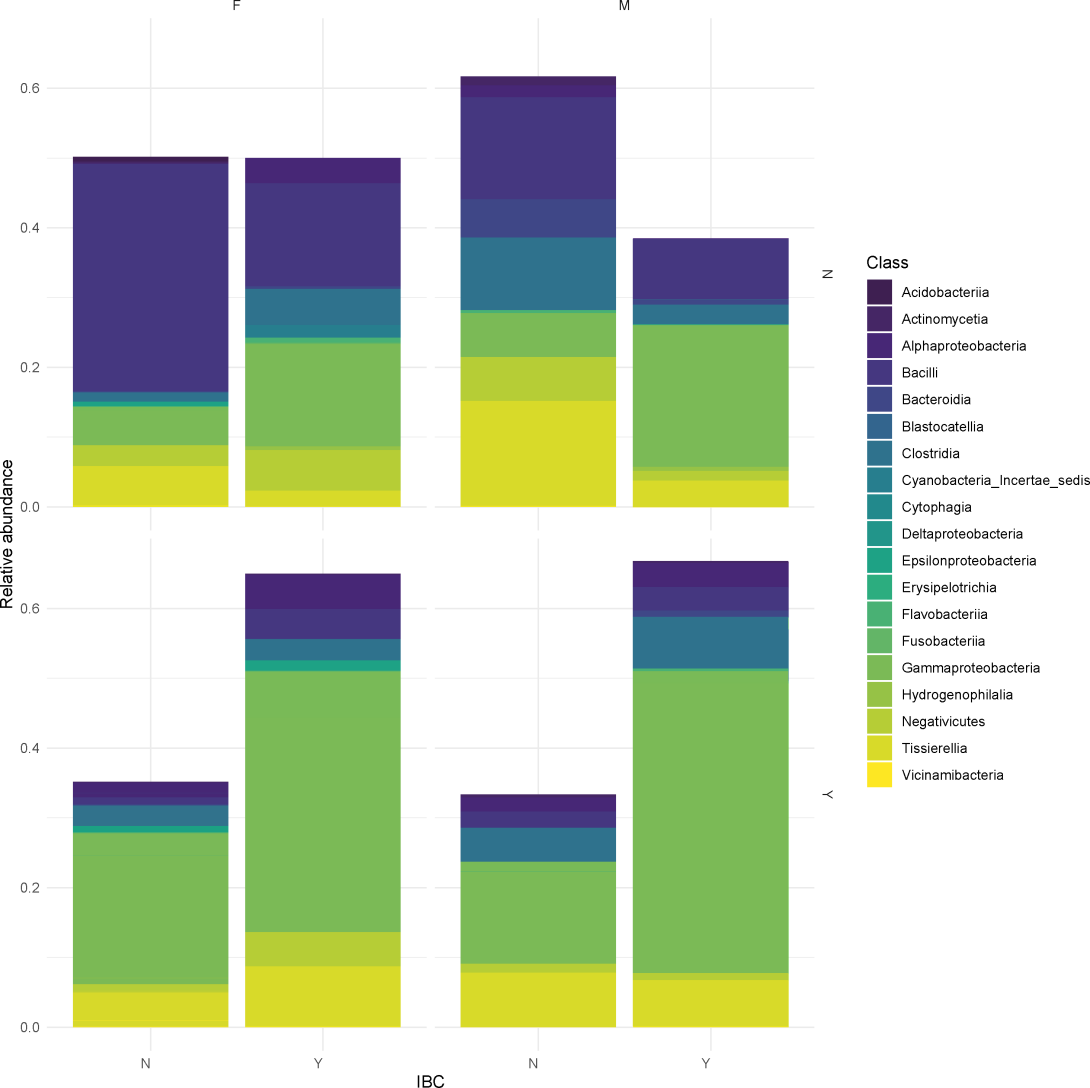
Urogenital microbiome composition across different variables, including sex, symptom presence, and intracellular bacterial communities. The stacked bar plots represent the relative abundance of the bacterial classes, with the colors corresponding to the taxonomic groups indicated in the legend.

Finally, bacterial class distributions according to sex, symptoms, and presence of IBC are shown in Fig. 7. The most significant differences in abundance were obtained at the class level (Table 2). Across all groups, *Actinobacteria*, *Gammaproteobacteria*, *Bacilli*, and *Bacteroidia* appear as the most abundant classes. Notably, males exhibit a higher relative abundance of *Actinobacteria*, whereas females show a more balanced distribution among multiple bacterial classes (i.e., *Bacilli* and *Gammaproteobacteria*).

**TABLE 2:** Summary of the differential abundance analysis (DAA) at the class level with the FDR-adjusted *P*-values

Comparisons	
Group 1	Group 2
Female asymptomatic: ↑ *Bacilli* (*P*-adj = 0.00007)	Male asymptomatic
Female asymptomatic with IBC: ↑ *Bacilli* (*P*-adj = 0.00063)	Male asymptomatic with IBC: ↑ *Gammaproteobacteria* (*P*-adj = 0.04940)
Female asymptomatic with IBC: ↑ *Gammaproteobacteria* (*P*-adj = 0.01174)	Female asymptomatic without IBC: ↑ *Bacilli* (*P*-adj = 0.00168)
Female symptomatic	Female asymptomatic: ↑ *Bacilli* (*P*-adj = 0.000002)
Male symptomatic: ↑ *Gammaproteobacteria* (*P*-adj = 0.00620)	Male asymptomatic

The symptomatic male population displayed a significantly increased abundance of *Gammaproteobacteria*, while the symptomatic female population displayed a significantly decreased abundance of *Bacilli* compared to asymptomatic individuals (Table 2), confirming the link between these bacterial communities and urinary tract conditions. Additionally, individuals with intracellular bacterial communities show variations in microbiome composition, with *Gammaproteobacteria* as the dominant taxa.

The core of microorganisms in the group of females without symptoms and absence of IBC is composed mainly of *Bacilli* (i.e., *Lactobacillus* spp. and *Staphylococcus* spp.) and, to a lesser extent, *Gammaproteobacteria* (i.e., *E. coli*). Even when symptoms or IBC appear, the proportion of *Gammaproteobacteria* increases considerably, and the amount of *Bacilli* decreases. In males, the same phenomena were also observed but with different genera in each class.

## DISCUSSION

Urinary tract infections (UTIs) represent one of the most common bacterial infections affecting mainly women, with nearly 50–60% experiencing at least one episode in their lifetime (13). The increased susceptibility in women is largely attributed to anatomical factors, such as a shorter urethra, proximity of the anus, sexual activity, hormonal changes, and pregnancy, among others. However, not all are supported by experimental evidence (14). The persistence of uropathogens within intracellular bacterial communities (IBC) and the disruption of protective microbial species, such as *Lactobacillus* spp., have been implicated in the pathogenesis of UTIs, highlighting the need for microbiome-targeted therapeutic strategies (15). Understanding the interactions between the urobiome and host defense mechanisms remains essential for developing effective, personalized approaches to UTI prevention and treatment. The paradigm of “urine sterility” has been changed in the last two decades by different findings, aligning with evidence supporting the presence of a diverse urobiome in healthy and symptomatic individuals (7, 16).

### Urogenital microbiome composition and sex differences

The method of urine sample collection can influence the composition of the urobiome and should, therefore, be taken into account when comparing different studies (8). In this study, samples were collected using the midstream technique; thus, the presence of microorganisms from the urethra and genital tract should be considered in the analysis of the urobiome composition. Different reports account for the presence of microorganisms from the skin in the male urobiome, while genital microorganisms appear in females (17). Although different microbiomes from various parts of the human body have been studied extensively, and it is known that they differ in many aspects, they cannot be regarded as completely independent (18). Moreover, it has been demonstrated that the different microbiotas communicate and act in a coordinated manner, ultimately influencing human well-being (18). In the UT, different variables, such as age, sex, and hormonal state, can affect the microbial composition of the urobiome.

In healthy populations of males and females, the urobiome is less abundant and diverse than other microbiomes (19). Differences in the urogenital microbiome composition between males and females, particularly in bacterial diversity and relative abundance, have been observed in the present report, although no significant differences were detected. While *E. coli* was found to be predominant in symptomatic females, symptomatic males exhibited a more heterogeneous microbiome, including *Musicola* spp., *Pantoea* spp., and *Anaerostipes* spp. These findings are consistent with previous studies showing sex-based microbiome variations likely influenced by hormonal factors and host immune responses (20, 21). Moreover, a higher richness of *Lactobacillus* spp. was exhibited by asymptomatic females, supporting their proposed protective role against uropathogens (22).

Despite these differences, age is another factor that could influence the urobiome composition. It has been shown that in males over 60 years of age and in postmenopausal females, changes in urobiome composition are observed (23). In healthy males, the presence of *Lactobacillus* spp., *Streptococcus* spp., *Corynebacterium* spp., *Staphylococcus* spp., and *Anaerococcus* spp. is reported as part of the male genitourinary microbiota (24). In contrast, in males with UTI, other microorganisms appear as *E. coli*, *Aerococcus urinae*, *Actinobaculum schaalii*, and *E. faecalis*, among others, and in the absence of *Lactobacilli* spp. (23–25). Interestingly, we were unable to detect or recover *Aerococcus* spp. and *Actinobaculum* spp. in the male UTI samples from our study. Regarding *Lactobacillus* spp., we observed their presence in elderly males; however, the size of this population was too small to draw strong conclusions. Additionally, our cohort had a median age of ~46, younger than the population studied in the referenced work (≥60 years). When we specifically re-examined sequencing data from the seven males >60 years old, we found no *Aerococcus* spp. or *Actinotignum* spp., and these genera were also absent in younger male participants.

In females, the proportion of beneficial microorganisms as *Lactobacillus* spp. decreased with menopause associated probably with the hormone change (3, 23, 26). It is worth noting that most urobiome studies have been done in Europe, USA, and China, and there are few reports evaluating the evolution of urobiome in Latin American females. Here, we described the composition of genitourinary microbiomes in only six postmenopausal females, 50% of whom presented UTI symptoms. Even though this is a small sample, we found that asymptomatic postmenopausal females had *L. crispatus* in urine. This finding should be interpreted with caution given the small sample size, but it could be related to geographical distribution or cultural differences.

### Role of *Lactobacilli* and *Staphylococcus* in urinary health

In the present work, *Lactobacillus* spp., particularly *L. crispatus* and *L. iners*, were highly abundant in asymptomatic females and less prevalent in symptomatic individuals or those with IBCs. In particular, *L. crispatus* was found to be associated with healthy females without symptoms and IBC. Additionally, EQUC enabled the recovery of this microorganism from urine, confirming its viability in the urine. *L. crispatus* has been reported as part of the urobiome and vaginal microbiome of healthy females (27). These results also support the idea that the origin of the urinary microbiome could be the other closest microbiota, such as the vaginal or intestinal microbiota, or even the skin (28), and more interesting microbiomes are connected (27). Here, we also observed the presence of *L. iners* in females with IBC and also in some males, all of them without symptoms. *L. iners* have been observed in healthy females, while other *Lactobacillus*, such as *L. gasseri*, has been observed in females with urinary incontinence (29). The hypothesis that *Lactobacilli* contribute to urinary tract health through competitive exclusion and antimicrobial production is reinforced by this finding (30).

Similarly, *Staphylococcus* spp., including *S. haemolyticus* and *S. epidermidis*, were commonly detected in both sexes, suggesting a commensal role that may shift under pathogenic conditions (31). Notably, these species were also detected intracellularly, raising questions about their potential role in persistent or recurrent infections. A set of diverse *Staphylococcus* species was observed in the present work and comprised the presence of 10 different cultivable species. Similar findings were also reported by other authors, but the relevance of these microorganisms in the urobiome remains unclear (32). Moreover, it has been recently reported that different staphylococci isolated from the skin can produce neurotransmitters (NT) (33). *S. epidermidis* is part of the normal skin microbiota, and the impact of its role in NT production needs to be addressed. Less is known about this ability of *Staphylococcus* spp. in the bladder, but it would be interesting to assess the potential of the different strains to produce NT and to modulate the brain-gut-bladder axis.

### Intracellular bacterial communities and their implications

The presence of IBCs was confirmed in nearly half of the study population, with higher detection in symptomatic individuals. In a previous work, a significant association was observed between IBC presence, symptoms of UTI, and female (6). Here, the association was not significant probably due to the population size. *E. coli* was the most frequently detected intracellular pathogen, particularly in symptomatic females, consistent with its known ability to form IBCs and contribute to UTI recurrence (6, 34, 35). Probably, this is a strategy employed by *E. coli* to survive in a complex and hostile environment like the bladder. The detection of other intracellular bacteria, like *S. maltophilia* and *S. haemolyticus*, suggests that IBC formation may not be exclusively associated with uropathogenic *E. coli* (UPEC), warranting further investigation into their pathogenic potential (6, 36). One relevant aspect is the presence of IBC in females without symptoms of UTI. In these cases, the urine microbiome could have a relevant role in controlling the intracellular bacteria and the potential development of UTI.

### Urogenital microbiome diversity and symptom status

Alpha diversity analysis revealed that symptomatic individuals exhibited greater microbial richness and diversity compared with asymptomatic individuals. These results contrast with previous studies in which UTI-associated dysbiosis was linked to reduced diversity (3, 37). However, the increased diversity in symptomatic individuals in this study may indicate a broader range of potential pathogens or a disrupted microbial community associated with infection. Interestingly, no significant differences in diversity were observed between individuals with and without IBCs, suggesting that intracellular colonization may not directly impact overall microbial richness but could play a role in pathogenesis through persistence mechanisms (15).

### Clinical implications and future directions

The growing need for microbiome-based diagnostics and new therapeutic approaches in UTI management is reinforced by these findings. Traditional culture methods often fail to detect the full diversity of urinary microbes, underestimating the presence of non-traditional uropathogens and potential protective species (16). Further research should focus on the functional roles of the urinary microbiome, particularly concerning host immunity and antimicrobial resistance. The observed sex-based differences also highlight the importance of personalized treatment strategies for UTIs, considering microbial composition and host factors.

New insights into the urinary microbiome and its relationship with urinary tract infections (UTIs), intracellular bacterial communities (IBCs), and sex-based differences have been provided by this study, which may affect understanding microbial dynamics in urinary health.

### Conclusion

The complexity of the urinary microbiome, its relationship with symptoms, and its role in recurrent infections have been underscored by this study. The differential presence of *Lactobacilli* and *Staphylococcus* spp. in asymptomatic and symptomatic individuals suggests potential protective mechanisms that warrant further exploration. Additionally, the detection of IBCs in multiple bacterial species raises new questions about their contribution to urinary pathophysiology. A better understanding of these interactions may lead to improved diagnostic and therapeutic strategies for UTIs, moving beyond the conventional pathogen-focused approach toward a more comprehensive view of urinary health.

## SUPPLEMENTAL MATERIAL

Supplemental tables spectrum.01247-25-s0001.docx Tables S1 to S3.

ASM does not own the copyrights to Supplemental Material that may be linked to, or accessed through, an article. The authors have granted ASM a non-exclusive, world-wide license to publish the Supplemental Material files. Please contact the corresponding author directly for reuse.

## References

[1] NIH HMP Working Group, Peterson J, Garges S, Giovanni M, McInnes P, Wang L, Schloss JA, Bonazzi V, McEwen JE, Wetterstrand KA, et al.. 2009. The NIH human microbiome project. Genome Res 19:2317–2323. doi:10.1101/gr.096651.109 19819907 PMC2792171

[2] Safarchi A, Al-Qadami G, Tran CD, Conlon M. 2025. Understanding dysbiosis and resilience in the human gut microbiome: biomarkers, interventions, and challenges. Front Microbiol 16:1559521. doi:10.3389/fmicb.2025.1559521 40104586 PMC11913848

[3] Whiteside SA, Razvi H, Dave S, Reid G, Burton JP. 2015. The microbiome of the urinary tract--a role beyond infection. Nat Rev Urol 12:81–90. doi:10.1038/nrurol.2014.361 25600098

[4] Pallares-Mendez R, Cervantes-Miranda DE, Gonzalez-Colmenero AD, Ochoa-Arvizo MA, Gutierrez-Gonzalez A. 2022. A perspective of the urinary microbiome in lower urinary tract infections - a review. Curr Urol Rep 23:235–244. doi:10.1007/s11934-022-01108-7 36053406

[5] Wojciuk B, Salabura A, Grygorcewicz B, Kędzierska K, Ciechanowski K, Dołęgowska B. 2019. Urobiome: in sickness and in health. Microorganisms 7:548. doi:10.3390/microorganisms7110548 31717688 PMC6921077

[6] Robino L, Sauto R, Morales C, Navarro N, González MJ, Cruz E, Neffa F, Zeballos J, Scavone P. 2024. Presence of intracellular bacterial communities in uroepithelial cells, a potential reservoir in symptomatic and non-symptomatic people. BMC Infect Dis 24:590. doi:10.1186/s12879-024-09489-5 38886658 PMC11181538

[7] Hilt EE, McKinley K, Pearce MM, Rosenfeld AB, Zilliox MJ, Mueller ER, Brubaker L, Gai X, Wolfe AJ, Schreckenberger PC. 2014. Urine is not sterile: use of enhanced urine culture techniques to detect resident bacterial flora in the adult female bladder. J Clin Microbiol 52:871–876. doi:10.1128/JCM.02876-13 24371246 PMC3957746

[8] Brubaker L, Gourdine J-PF, Siddiqui NY, Holland A, Halverson T, Limeria R, Pride D, Ackerman L, Forster CS, Jacobs KM, Thomas-White KJ, Putonti C, Dong Q, Weinstein M, Lukacz ES, Karstens L, Wolfe AJ. 2021. Forming consensus to advance urobiome research. mSystems 6:e0137120. doi:10.1128/mSystems.01371-20 34282932 PMC8409733

[9] Oksanen J, Simpson GL, Blanchet FG, Kindt R, Legendre P, Minchin P, O’Hara R, Solymos P, Stevens M, Szoecs E, et al.. 2022. vegan: community ecology package (version 2.6-4). Available from: https://cran.r-project.org/web/packages/vegan/index.html

[10] Love MI, Huber W, Anders S. 2014. Moderated estimation of fold change and dispersion for RNA-seq data with DESeq2. Genome Biol 15:550. doi:10.1186/s13059-014-0550-8 25516281 PMC4302049

[11] Peschel S, Müller CL, von Mutius E, Boulesteix A-L, Depner M. 2021. NetCoMi: network construction and comparison for microbiome data in R. Brief Bioinform 22:bbaa290. doi:10.1093/bib/bbaa290 33264391 PMC8293835

[12] McMurdie PJ, Holmes S. 2013. Phyloseq: an R package for reproducible interactive analysis and graphics of microbiome census data. PLoS One 8:e61217. doi:10.1371/journal.pone.0061217 23630581 PMC3632530

[13] Flores-Mireles AL, Walker JN, Caparon M, Hultgren SJ. 2015. Urinary tract infections: epidemiology, mechanisms of infection and treatment options. Nat Rev Microbiol 13:269–284. doi:10.1038/nrmicro3432 25853778 PMC4457377

[14] Deltourbe L, Lacerda Mariano L, Hreha TN, Hunstad DA, Ingersoll MA. 2022. The impact of biological sex on diseases of the urinary tract. Mucosal Immunol 15:857–866. doi:10.1038/s41385-022-00549-0 35869147 PMC9305688

[15] Brubaker L, Wolfe AJ. 2015. The new world of the urinary microbiota in women. Am J Obstet Gynecol 213:644–649. doi:10.1016/j.ajog.2015.05.032 26003055 PMC4876712

[16] Price TK, Dune T, Hilt EE, Thomas-White KJ, Kliethermes S, Brincat C, Brubaker L, Wolfe AJ, Mueller ER, Schreckenberger PC. 2016. The clinical urine culture: enhanced techniques improve detection of clinically relevant microorganisms. J Clin Microbiol 54:1216–1222. doi:10.1128/JCM.00044-16 26962083 PMC4844725

[17] Romain SR. 2023. The urobiome in men and women: a clinical review. Clin Microbiol Infect 29:1242–1248. doi:10.1016/j.cmi.2022.08.010 36028087

[18] Martínez JE, Vargas A, Pérez-Sánchez T, Encío IJ, Cabello-Olmo M, Barajas M. 2021. Human microbiota network: unveiling potential crosstalk between the different microbiota ecosystems and their role in health and disease. Nutrients 13:2905. doi:10.3390/nu13092905 34578783 PMC8466470

[19] Chorbińska J, Krajewski W, Nowak Ł, Małkiewicz B, Del Giudice F, Szydełko T. 2023. Urinary microbiome in bladder diseases-review. Biomedicines 11:2816. doi:10.3390/biomedicines11102816 37893189 PMC10604329

[20] Kogan MI, Naboka YL, Ibishev KS, Gudima IA, Naber KG. 2021. Human urine is not sterile—shift of paradigm. Urol Int 105:356–368. doi:10.1159/000369631 25766599

[21] Gottschick C, Deng Z, Vital M, Masur C, Abels C, Pieper H, Wagner-Döbler I. 2017. The urinary microbiome of men and women and its changes in urinary tract infections. Microbiome 5:78. doi:10.1186/s40168-017-0305-3 28807017 PMC5554977

[22] Stapleton AE. 2016. The vaginal microbiota and urinary tract infection. Microbiol Spectr 4. doi:10.1128/microbiolspec.UTI-0025-2016 PMC5746606 28087949

[23] Qin J, Shi X, Xu J, Yuan S, Zheng B, Zhang E, Huang G, Li G, Jiang G, Gao S, Tian C, Guo R, Fu Z, Huang Q, Yang R, Zhang W, Li S, Wu S. 2021. Characterization of the genitourinary microbiome of 1,165 middle-aged and elderly healthy individuals. Front Microbiol 12:673969. doi:10.3389/fmicb.2021.673969 34489882 PMC8417382

[24] Roth RS, Liden M, Huttner A. 2023. The urobiome in men and women: a clinical review. Clin Microbiol Infect 29:1242–1248. doi:10.1016/j.cmi.2022.08.010 36028087

[25] Haley E, Luke N, Mathur M, Festa RA, Wang J, Jiang Y, Anderson LA, Baunoch D. 2024. The Prevalence and association of different uropathogens detected by M-PCR with infection-associated urine biomarkers in urinary tract infections. Res Rep Urol 16:19–29. doi:10.2147/RRU.S443361 38221993 PMC10787514

[26] Tang J. 2017. Microbiome in the urinary system-a review. AIMS Microbiol 3:143–154. doi:10.3934/microbiol.2017.2.143 31294154 PMC6605016

[27] Ferneyhough B, Roddis M, Millington S, Quirk J, Clements C, West S, Schilizzi R, Fischer MD, Parkinson NJ. 2025. A highly accurate nanopore-based sequencing workflow for culture and PCR-free microbial metagenomic profiling of urogenital samples. BMC Urol 25:41. doi:10.1186/s12894-025-01723-9 40022097 PMC11869423

[28] Čeprnja M, Hadžić E, Oros D, Melvan E, Starcevic A, Zucko J. 2023. Current viewpoint on female urogenital microbiome-the cause or the consequence? Microorganisms 11:1207. doi:10.3390/microorganisms11051207 37317181 PMC10224287

[29] Pearce MM, Hilt EE, Rosenfeld AB, Zilliox MJ, Thomas-White K, Fok C, Kliethermes S, Schreckenberger PC, Brubaker L, Gai X, Wolfe AJ. 2014. The female urinary microbiome: a comparison of women with and without urgency urinary incontinence. mBio 5:e01283-14. doi:10.1128/mBio.01283-14 25006228 PMC4161260

[30] Vazquez-Munoz R, Dongari-Bagtzoglou A. 2021. Anticandidal Activities by Lactobacillus species: an update on mechanisms of action. Front Oral Health 2:689382. doi:10.3389/froh.2021.689382 35048033 PMC8757823

[31] Davis NM, Proctor DM, Holmes SP, Relman DA, Callahan BJ. 2018. Simple statistical identification and removal of contaminant sequences in marker-gene and metagenomics data. Microbiome 6:226. doi:10.1186/s40168-018-0605-2 30558668 PMC6298009

[32] Kenneally C, Murphy CP, Sleator RD, Culligan EP. 2022. The urinary microbiome and biological therapeutics: novel therapies for urinary tract infections. Microbiol Res 259:127010. doi:10.1016/j.micres.2022.127010 35338973

[33] Rahmdel S, Purkayastha M, Nega M, Liberini E, Li N, Luqman A, Brüggemann H, Götz F. 2024. Diversity of neurotransmitter-producing human skin commensals. Int J Mol Sci 25:12345. doi:10.3390/ijms252212345 39596410 PMC11595044

[34] Mulvey MA, Schilling JD, Hultgren SJ. 2001. Establishment of a persistent Escherichia coli reservoir during the acute phase of a bladder infection. Infect Immun 69:4572–4579. doi:10.1128/IAI.69.7.4572-4579.2001 11402001 PMC98534

[35] Robino L, Scavone P, Araujo L, Algorta G, Zunino P, Pírez MC, Vignoli R. 2014. Intracellular bacteria in the pathogenesis of Escherichia coli urinary tract infection in children. Clin Infect Dis 59:e158–64. doi:10.1093/cid/ciu634 25091303 PMC4650771

[36] Mysorekar IU, Hultgren SJ. 2006. Mechanisms of uropathogenic Escherichia coli persistence and eradication from the urinary tract. Proc Natl Acad Sci USA 103:14170–14175. doi:10.1073/pnas.0602136103 16968784 PMC1564066

[37] Fouts DE, Pieper R, Szpakowski S, Pohl H, Knoblach S, Suh MJ, Huang ST, Ljungberg I, Sprague BM, Lucas SK, Torralba M, Nelson KE, Groah SL. 2012. Integrated next-generation sequencing of 16S rDNA and metaproteomics differentiate the healthy urine microbiome from asymptomatic bacteriuria in neuropathic bladder associated with spinal cord injury. J Transl Med 10:174. doi:10.1186/1479-5876-10-174 22929533 PMC3511201

