# Supplementary material for: Urogenital microbiome, intracellular bacterial communities, and their contribution to urinary tract infections
Source: Microbiol Spectr. 2025 Oct 7;13(11):e01247-25. doi: 10.1128/spectrum.01247-25 (PMC12584659; doi:10.1128/spectrum.01247-25)
Supplement: Supplemental tables — Tables S1 to S3. [file spectrum.01247-25-s0001.docx]

Supplementary material

**Table S1**: Intracellular bacteria distribution among the different groups. The X2 statistical test was applied to each group (female and male) in order to evaluate significant association with symptoms and IBC presence. P was considered significant when P<0.05.

|  | Intracellular bacteria | | | |
| --- | --- | --- | --- | --- |
|  | Presence (N=37) | Absence (N=40) | Total | P-value |
| Female with symptoms (N=22) | 14 | 8 | 22 | 0.38 |
| Female non-symptoms (N=18) | 9 | 9 | 18 |  |
| Male with symptoms (N=17) | 9 | 8 | 17 | 0.08 |
| Male non-symptoms (N=20) | 5 | 15 | 20 |  |

**Table S2**: Abundance of the top 10 different genus according to the different group analyzed (All females, females without symptoms, females with symptoms and females postmenopausal)

| All Females | |  |  |  |  |
| --- | --- | --- | --- | --- | --- |
| tax_rank | abundance | class | order | family | genus |
| 1 | 0,21643528 | Bacilli | Lactobacillales | Lactobacillaceae | Lactobacillus |
| 2 | 0,0961957 | Gammaproteobacteria | Burkholderiales | Burkholderiaceae | Ralstonia |
| 3 | 0,093644 | Gammaproteobacteria | Enterobacterales | Enterobacteriaceae | Escherichia |
| 4 | 0,06070744 | Gammaproteobacteria | Xanthomonadales | Xanthomonadaceae | Stenotrophomonas |
| 5 | 0,05264475 | Gammaproteobacteria | Burkholderiales | Alcaligenaceae | Achromobacter |
| 6 | 0,03830166 | Tissierellia | Tissierellales | Peptoniphilaceae | Anaerococcus |
| 7 | 0,0365793 | Gammaproteobacteria | Burkholderiales | Burkholderiaceae | Cupriavidus |
| 8 | 0,03441164 | Tissierellia | Tissierellales | Peptoniphilaceae | Peptoniphilus |
| 9 | 0,0305506 | Bacilli | Bacillales | Bacillaceae | Bacillus |

| Females without Symptoms | | |  |  |  |
| --- | --- | --- | --- | --- | --- |
| tax_rank | abundance | class | order | family | genus |
| 1 | 0,38632183 | Bacilli | Lactobacillales | Lactobacillaceae | Lactobacillus |
| 2 | 0,10832596 | Gammaproteobacteria | Burkholderiales | Burkholderiaceae | Ralstonia |
| 3 | 0,07116189 | Gammaproteobacteria | Xanthomonadales | Xanthomonadaceae | Stenotrophomonas |
| 4 | 0,04492611 | Tissierellia | Tissierellales | Peptoniphilaceae | Anaerococcus |
| 5 | 0,03704703 | Tissierellia | Tissierellales | Peptoniphilaceae | Peptoniphilus |
| 6 | 0,03450194 | Gammaproteobacteria | Enterobacterales | Enterobacteriaceae | Escherichia |
| 7 | 0,03089032 | Gammaproteobacteria | Enterobacterales | Morganellaceae | Proteus |
| 8 | 0,02280868 | Bacilli | Lactobacillales | Streptococcaceae | Streptococcus |
| 9 | 0,01904762 | Alphaproteobacteria | Hyphomicrobiales | Phyllobacteriaceae | Phyllobacterium |
| 10 | 0,01851852 | Cyanobacteria_Incertae_sedis | Pseudanabaenales | Oculatellaceae | Timaviella |

| Females with Symptoms | |  |  |  |  |
| --- | --- | --- | --- | --- | --- |
| tax_rank | abundance | class | order | family | genus |
| 1 | 0,13634 | Gammaproteobacteria | Enterobacterales | Enterobacteriaceae | Escherichia |
| 2 | 0,08874459 | Gammaproteobacteria | Burkholderiales | Alcaligenaceae | Achromobacter |
| 3 | 0,08627095 | Gammaproteobacteria | Burkholderiales | Burkholderiaceae | Ralstonia |
| 4 | 0,0774372 | Bacilli | Lactobacillales | Lactobacillaceae | Lactobacillus |
| 5 | 0,0655303 | Gammaproteobacteria | Burkholderiales | Burkholderiaceae | Cupriavidus |
| 6 | 0,05270563 | Bacilli | Bacillales | Bacillaceae | Bacillus |
| 7 | 0,0521538 | Gammaproteobacteria | Xanthomonadales | Xanthomonadaceae | Stenotrophomonas |
| 8 | 0,04567656 | Bacteroidia | Bacteroidales | Prevotellaceae | Prevotella |
| 9 | 0,03288165 | Tissierellia | Tissierellales | Peptoniphilaceae | Anaerococcus |

| Females postmenopausal | | |  |  |  |
| --- | --- | --- | --- | --- | --- |
| tax_rank | abundance | class | order | family | genus |
| 1 | 0,20593027 | Gammaproteobacteria | Burkholderiales | Burkholderiaceae | Ralstonia |
| 2 | 0,19265233 | Gammaproteobacteria | Xanthomonadales | Xanthomonadaceae | Stenotrophomonas |
| 3 | 0,166062 | Gammaproteobacteria | Enterobacterales | Enterobacteriaceae | Escherichia |
| 4 | 0,11516536 | Bacilli | Lactobacillales | Lactobacillaceae | Lactobacillus |
| 5 | 0,09848485 | Gammaproteobacteria | Burkholderiales | Alcaligenaceae | Achromobacter |
| 6 | 0,04791667 | Bacteroidia | Bacteroidales | Prevotellaceae | Prevotella |
| 7 | 0,03260279 | Gammaproteobacteria | Enterobacterales | Enterobacteriaceae | Enterobacter |
| 8 | 0,03030303 | Clostridia | Eubacteriales | Lachnospiraceae | Mediterraneibacter |
| 9 | 0,03030303 | Gammaproteobacteria | Burkholderiales | Burkholderiaceae | Paraburkholderia |

**Table S3**: Abundance of the top 10 different genus according to the different group analyzed (All males, males without symptoms, males with symptoms and elder males).

| All Males |  |  |  |  |  |
| --- | --- | --- | --- | --- | --- |
| tax_rank | abundance | class | order | family | genus |
| 1 | 0,16464962 | Bacilli | Lactobacillales | Lactobacillaceae | Lactobacillus |
| 2 | 0,1235 | Gammaproteobacteria | Enterobacterales | Enterobacteriaceae | Escherichia |
| 3 | 0,09628838 | Gammaproteobacteria | Xanthomonadales | Xanthomonadaceae | Stenotrophomonas |
| 4 | 0,07871246 | Gammaproteobacteria | Burkholderiales | Burkholderiaceae | Ralstonia |
| 5 | 0,05449357 | Gammaproteobacteria | Burkholderiales | Alcaligenaceae | Achromobacter |
| 6 | 0,03647575 | Bacilli | Bacillales | Bacillaceae | Bacillus |
| 7 | 0,03634767 | Alphaproteobacteria | Hyphomicrobiales | Phyllobacteriaceae | Phyllobacterium |
| 8 | 0,03088803 | Alphaproteobacteria | Rhodospirillales | Rhodospirillaceae | Taonella |
| 9 | 0,02856298 | Gammaproteobacteria | Enterobacterales | Enterobacteriaceae | Klebsiella |

| Males without Symptoms | | |  |  |  |
| --- | --- | --- | --- | --- | --- |
| tax_rank | abundance | class | order | family | genus |
| 1 | 0,14421219 | Bacilli | Lactobacillales | Lactobacillaceae | Lactobacillus |
| 2 | 0,10241907 | Gammaproteobacteria | Xanthomonadales | Xanthomonadaceae | Stenotrophomonas |
| 3 | 0,08771416 | Gammaproteobacteria | Enterobacterales | Enterobacteriaceae | Escherichia |
| 4 | 0,0803785 | Gammaproteobacteria | Burkholderiales | Alcaligenaceae | Achromobacter |
| 5 | 0,05714286 | Alphaproteobacteria | Rhodospirillales | Rhodospirillaceae | Taonella |
| 6 | 0,05519481 | Alphaproteobacteria | Hyphomicrobiales | Phyllobacteriaceae | Phyllobacterium |
| 7 | 0,05 | Gammaproteobacteria | Burkholderiales | Burkholderiaceae | Paraburkholderia |
| 8 | 0,05 | Gammaproteobacteria | Enterobacterales | Enterobacteriaceae | Klebsiella |
| 9 | 0,05 | Bacteroidia | Bacteroidales | Rikenellaceae | Millionella |

| Males with Symptoms | |  |  |  |  |
| --- | --- | --- | --- | --- | --- |
| tax_rank | abundance | class | order | family | genus |
| 1 | 0,18869366 | Bacilli | Lactobacillales | Lactobacillaceae | Lactobacillus |
| 2 | 0,17353 | Gammaproteobacteria | Enterobacterales | Enterobacteriaceae | Escherichia |
| 3 | 0,13662635 | Gammaproteobacteria | Burkholderiales | Burkholderiaceae | Ralstonia |
| 4 | 0,08907581 | Gammaproteobacteria | Xanthomonadales | Xanthomonadaceae | Stenotrophomonas |
| 5 | 0,04298053 | Bacilli | Bacillales | Bacillaceae | Bacillus |
| 6 | 0,03878844 | Epsilonproteobacteria | Campylobacterales | Campylobacteraceae | Campylobacter |
| 7 | 0,02511505 | Gammaproteobacteria | Burkholderiales | Burkholderiaceae | Cupriavidus |
| 8 | 0,02404072 | Gammaproteobacteria | Burkholderiales | Alcaligenaceae | Achromobacter |
| 9 | 0,01544031 | Bacteroidia | Bacteroidales | Porphyromonadaceae | Porphyromonas |

| Males Elders | |  |  |  |  |
| --- | --- | --- | --- | --- | --- |
| tax_rank | abundance | class | order | family | genus |
| 1 | 0,23737205 | Bacilli | Lactobacillales | Lactobacillaceae | Lactobacillus |
| 2 | 0,16824319 | Gammaproteobacteria | Burkholderiales | Burkholderiaceae | Ralstonia |
| 3 | 0,13246 | Gammaproteobacteria | Enterobacterales | Enterobacteriaceae | Escherichia |
| 4 | 0,07097374 | Bacilli | Lactobacillales | Streptococcaceae | Streptococcus |
| 5 | 0,03760189 | Gammaproteobacteria | Burkholderiales | Burkholderiaceae | Cupriavidus |
| 6 | 0,02285712 | Bacilli | Bacillales | Bacillaceae | Bacillus |
| 7 | 0,01998112 | Gammaproteobacteria | Xanthomonadales | Xanthomonadaceae | Stenotrophomonas |
| 8 | 0,0162296 | Gammaproteobacteria | Enterobacterales | Enterobacteriaceae | Citrobacter |
| 9 | 0,01573427 | Tissierellia | Tissierellales | Peptoniphilaceae | Anaerococcus |
